# Physiological and Perceptual Responses to Nordic Walking in a Natural Mountain Environment

**DOI:** 10.3390/ijerph14101235

**Published:** 2017-10-17

**Authors:** Alessandro Grainer, Livio Zerbini, Carlo Reggiani, Giuseppe Marcolin, James Steele, Gaspare Pavei, Antonio Paoli

**Affiliations:** 1 Department of Biomedical Sciences, University of Padova, Via Marzolo, 3, 35131 Padova, Italy; supergrainer@gmail.com (A.G.); carlo.reggiani@unipd.it (G.R.); giuseppe.marcolin@unipd.it (G.M.); 2 CeRiSM, Sport Mountain Health Research Center, 38068 Rovereto, Italy; livio.zerbini@gmail.com; 3 Centre for Health, Exercise and Sport Science, Southampton Solent University, Southampton SO14 0YN, UK; james.steele@solent.ac.uk; 4 Department of Pathophysiology and Transplantation, University of Milan, 20122 Milan, Italy; gaspare.pavei@unimi.it

**Keywords:** pole walking locomotion, energy expenditure, RPE, natural environment, trekking

## Abstract

*Background:* Interest around Nordic Walking (NW) has increased in recent years. However, direct comparisons of NW with normal walking (W), particularly in ecologically valid environments is lacking. The aim of our study was to compare NW and W, over long distances in a natural mountain environment. *Methods:* Twenty one subjects (13 male/8 female, aged 41 ± 12 years, body mass index BMI 24.1 ± 3.7), walked three distinct uphill paths (length 2.2/3.4/7 km) with (NW) or without (W) walking poles over two separate days. Heart rate (HR), energy expenditure (EE), step length (SL), walking speed (WS), total steps number (SN) and rating of perceived exertion (RPE) were monitored. *Results:* HR (+18%) and EE (+20%) were higher in NW than in W whilst RPE was similar. SN (−12%) was lower and SL (+15%) longer in NW. WS was higher (1.64 vs. 1.53 m s^−1^) in NW. *Conclusions:* Our data confirm that, similarly to previous laboratory studies, differences in a range of walking variables are present between NW and W when performed in a natural environment. NW appears to increase EE compared to W, despite a similar RPE. Thus, NW could be a useful as aerobic training modality for weight control and cardiorespiratory fitness.

## 1. Introduction

Walking is a basic motor skill, which can provide a pleasurable and rewarding physical activity in everyday life [1,2]. Nordic Walking (NW) is a four limb form of locomotion that involves the upper limbs through the use of poles, thus increasing the number of muscles activated [3] particularly those of the upper extremities [4]. In recent years, Nordic Walking has become increasingly popular because it allows performance of physical activity requiring a higher energy expenditure and an unaltered or diminished rating of perceived exertion (RPE) compared to ordinary walking (W) [5,6]. This makes NW particularly attractive for people interested in weight loss [5]. Moreover, the use of poles in NW has been proposed as a way to reduce the load on knees in comparison to ordinary W, though there are contrasting opinions regarding this issue [7,8].

For these reasons, NW has been promoted as an approach for the prevention of several diseases and to be included in the rehabilitation process. In fact, regular practise of NW has been reported as beneficial for chronic low back pain [9], osteoporosis [10], in rehabilitation after both coronary disease [11] and breast cancer [12], intermittent claudication [13], and in participants affected by Parkinson’s [14].

Despite the wealth of evidence regarding energy consumption during NW training sessions [3,5,6,7,15,16,17,18], it would appear that no studies have investigated the physiological and perceptual response of NW compared to W in a natural environment. Unlike in controlled laboratory conditions, a natural environment provides tracks with variable slopes and undulating surfaces of different terrain that may affect any differences in the physiological and perceptual responses between NW and W.

Thus in this study we aimed to assess the differences between NW and W including walking mechanics, heart rate, energy consumption, and rating of perceived effort in a group of participants following the same path in a mountain environment with poles (NW) and without poles (W). 

## 2. Materials and Methods

### 2.1. Participant Characteristics

A group of 21 participants (13 male/8 female, age 41 ± 12, body mass index BMI 24.1 ± 3.7) were recruited, all of whom were certified Nordic Walking instructors to ensure consistent and repeatable technical execution. All 21 participants took part in the first test of the study, while a subgroup of 8 participants (4 male/4 female, age 39 ± 10, BMI 23 ± 5.5) participated in the second and third test of the study. All participants were physically active and currently engaged in regular nordic walking (NW) and ordinary walking (W). Informed consent was obtained from all participants and the experimental protocol was approved by local Ethical Committee (CEIAF 3/11).

### 2.2. Research Design

**Figure 1:**
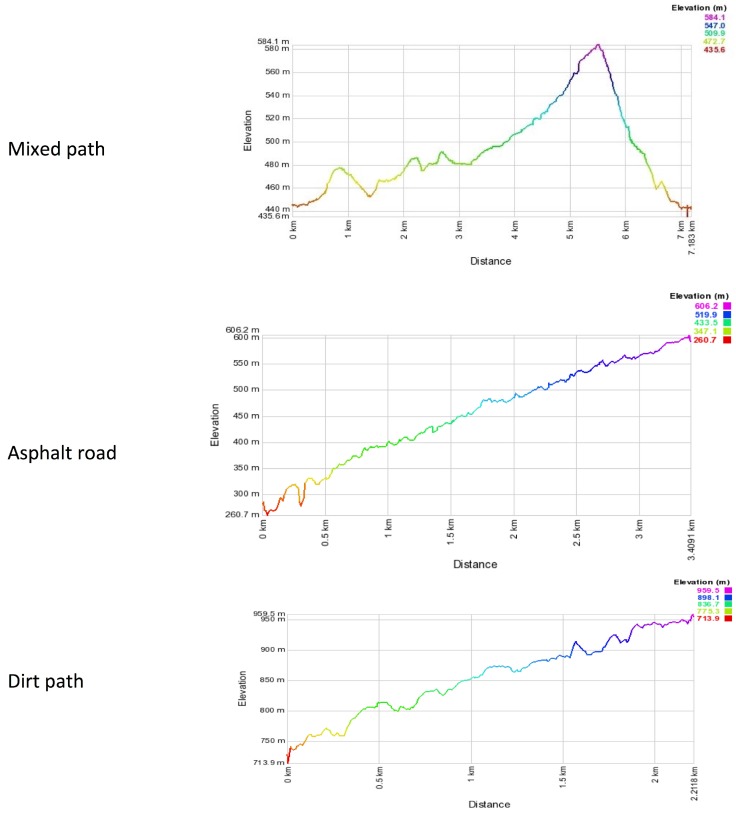
Elevation profiles of the three different experimental paths.

The study was based on three separate tests (see Figure 1) with each performed in two sessions under different conditions: one NW with poles and one ordinary W without poles. The sessions were randomly assigned, and were performed at the same time of the day (from 08:00 to 11:00 am) and in the same weather conditions (temperature from 18 to 21 °C, partly cloudy, humidity from 60 to 70%). 

The three tests involved performance of NW and ordinary W over three different paths. The first path was on a “mixed terrain surface”, over the distance of 7.2 km with an elevation gain of 150 m. The second and third paths were two climbs with a constant slope of 10%. The first climb had an “asphalt surface” whereas the second climb was a typical “off-road” track. Both were performed first uphill and then downhill at a self-selected speed. The choice of the two ramps of test 2 and 3 was in order to examine data from constant slopes and terrains, in contrast with the first track which represented an ecologically valid route. 

In order to provide further ecological validity with respect to performance of NW [19] participants were allowed to use their preferred poles and to walk at a self-selected speed [20]. This is due to it being difficult to maintain a given constant speed outdoor with different slopes and terrain both uphill and downhill, and particularly when performing a technical activity like NW [21]. 

### 2.3. Measurement Energy Expenditure and Locomotion Parameters

To reproduce as closely as possible the conditions of ordinary practise of NW, an indirect approach was selected to monitor energy consumption. Participants wore an Armband^®^ Sensewear (BodyMedia Inc., Pittsburgh, PA, USA), on the triceps muscle of the right arm, which enabled monitoring of metabolic responses during an activity lasting more than one hour without impediment to the performance of the activity. The armband measured number of steps and from this estimated energy expenditure. Armbands are an indirect method to monitor energy consumption, with well-known limitations such as a certain level of inaccuracy for cycling and during running in endurance athletes [22], but they can provide valid estimations of physical activity during low intensity activities such as Nordic Walking [23]. Careful calibration of the Armband^®^ Sensewear with reference to a standard expired gas analyser was performed prior to data collection. Subjects were asked to walk at 5.0, 5.5, and 6.0 km/h on a treadmill, at 0%, 3%, 5%, and 7.0% positive elevation, each for a duration of 5 min. The results obtained demonstrated that the agreement was good in the range of energy consumption rate (4–6 kcal/min) corresponding to NW on the mountain path and validated the indirect approach to measure energy consumption. A further important issue, which needs to be considered for the experimental design, is that NW is not a natural way of locomotion as W, but requires learning to obtain an effective total body exercise. For this reason, in this study experienced instructors were asked to participate to the study and to guarantee that the movement was performed in the optimal way. HR was monitored by a Polar^®^ s725x heart rate monitor (Polar, Kempele, Finland) and averaged across each of the trials.

### 2.4. Determination of Rating of Perceived Exertion (RPE)

RPE was assessed using the Borg scale (from 6 to 20) and administered with the appropriate questionnaire [24]. RPE was collected from participants both at the end of the uphill phase and at the end of the downhill phase of the second and third test.

### 2.5. Statistical Analysis

Descriptive data are expressed as means and standard deviations. The software package GraphPad Prism version 4.00 for Windows (GraphPad Software, San Diego, CA, USA) was used for regression analysis and student *t* test comparing W and NW. Alpha significance level was set at 0.05.

## 3. Results

### 3.1. Energy Ependiture of Locomotion 

**Figure 2:**
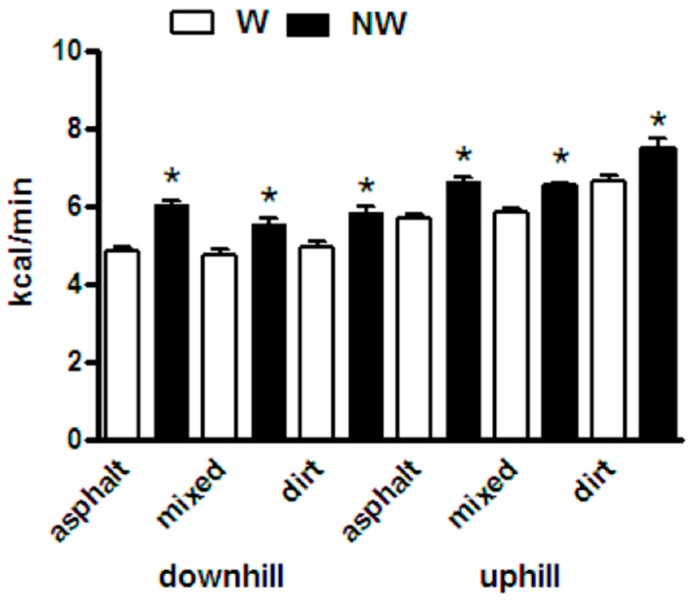
Average of the Energy Consumption Estimation for the “mixed terrain path”, “the asphalt path” and the “off-road path”. If the grand means (mean of the means) were compared, the cost of locomotion was 19.5% higher with poles (nordic walking NW, 6.57 ± 0.54 kcal min^−1^) than without poles (ordinary walking W, 5.49 ± 0.47 kcal min^−1^).

Figure 2 shows the average of the energy consumption estimations for the “mixed terrain path”, “the asphalt path” and the “off-road path”. For all the conditions the energy expenditure was significantly higher in NW respect to W (*p* < 0.05).

When the grand means (mean of the means) were compared, the cost of locomotion was 19.5% *p* < 0.001 higher with poles (NW, 6.57 ± 0.54 kcal min^-1^) than without poles (W, 5.49 ± 0.47 kcal min^−1^).

### 3.2. Heart Rate 

The average of all measurements from the three tests gave a higher heart rate value of 129 ± 19 beats min^−1^ for NW and a lower value of 108 ± 17 beats min^−1^ for W (+19%, *p* < 0.001). The paths were further subdivided in distinct segments, partly uphill and partly downhill, where HR values were recorded.

**Figure 3:**
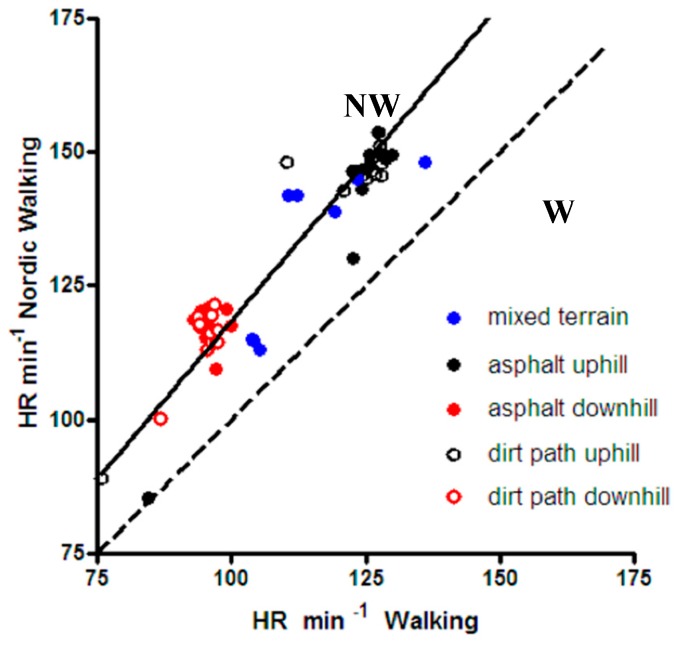
Average heart rates of all subjects recorded during NW (continuous line) and W (dashed line) sessions in each path segment. As expected, heart rate (HR) was higher when hiking uphill than downhill, but in both cases it was higher in NW than in W. The slope of the regression line (continuous line) of the values recorded in NW vs. the values recorded in W had a value of 1.184 ± 0.007, significantly greater than 1 (dashed line), *p* < 0.001.

Figure 3 compares the average heart rates of all subjects recorded during NW and W sessions in each path segment. As expected, heart rate was higher when hiking uphill than downhill, but in both cases it was higher in NW than in W. The slope of the regression line (continuous line in Figure 2) of the values recorded in NW vs. the values recorded in W had a value of 1.184 ± 0.007, significantly greater than 1 (dashed line), *p* < 0.001.

### 3.3. Locomotion Dynamics

**Figure 4:**
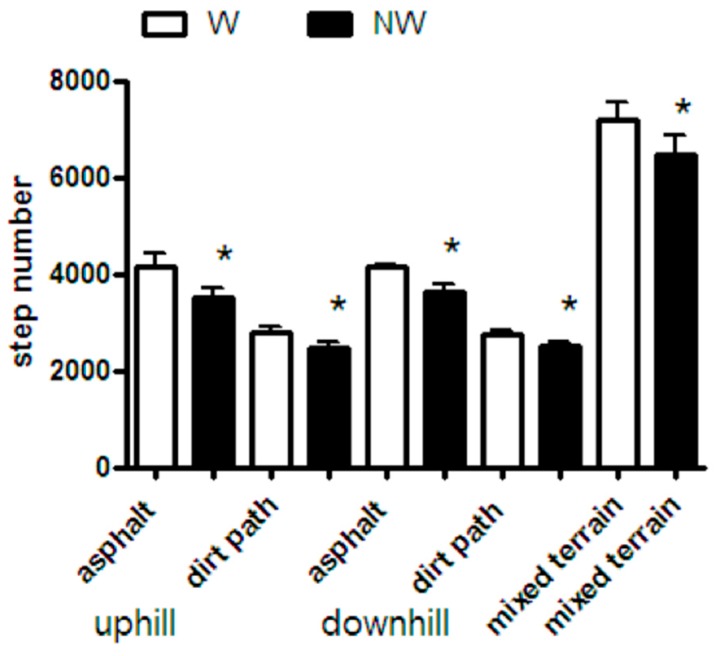
Average numbers of steps in the three tests are plotted for NW and W. The difference in step number implies a difference in stride length, which was calculated to be on average 0.92 ± 0.05 m in NW compared to 0.80 ± 0.04 m in W, using data of all subjects in the three tests (*p* < 0.001). In detail, average stride length on asphalt road (second test) was 0.82 ± 0.05 m in W and 0.97 ± 0.05 m in NW walking uphill and 0.82 ± 0.03 m in W and 0.93 ± 0.04 m in NW walking dowhill; on dirt path (third test) stride length was 0.79 ± 0.03 m in W and 0.88 ± 0.03 m in NW uphill and 0.77 ± 0.03 m in W and 0.88 ± 0.03 m in NW downhill.

For all the conditions the average speed was higher (+7.2%) in NW than in W (5.9 ± 0.3 vs. 5.5 ± 0.2 km h^−1^, *p* < 0.001) and the number of steps were lower (−15% *p* < 0.001) in NW than in W (*p* < 0.05). In Figure 4, the average numbers of steps in across all paths examined are presented. These suggest a greater stride length in the NW condition; calculated to be on average 0.92 ± 0.05 m in NW compared to 0.80 ± 0.04 m in W (*p* < 0.001). For each of the paths average stride lengths were as follows: asphalt road (second test) was 0.82 ± 0.05 m in W and 0.97 ± 0.05 m in NW walking uphill and 0.82 ± 0.03 m in W and 0.93 ± 0.04 m in NW walking downhill; on the dirt path (third test) stride length was 0.79 ± 0.03 m in W and 0.88 ± 0.03 m in NW uphill and 0.77 ± 0.03 m in W and 0.88 ± 0.03 m in NW downhill. 

### 3.4. Rating of Perceived Exertion

**Figure 5:**
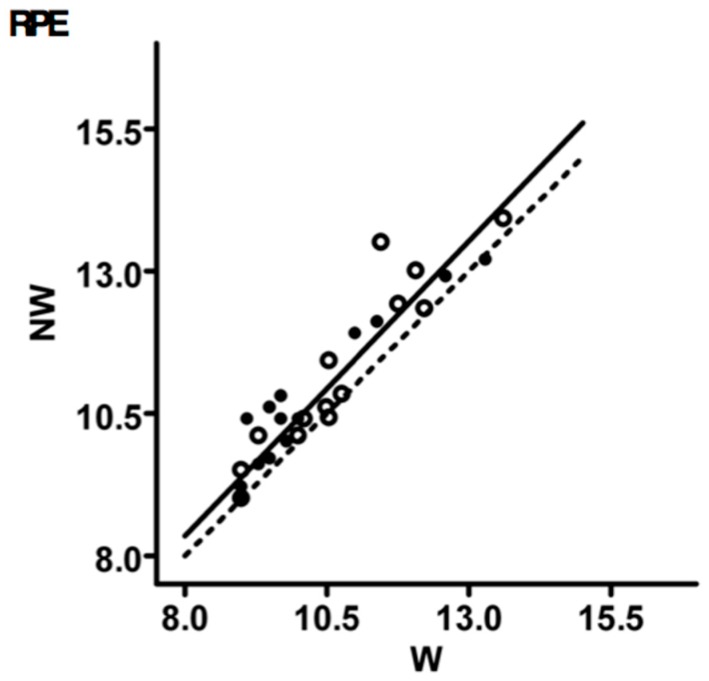
Perceived Exertion Intensity The regression line was slightly but not significantly above the 1:1 correspondence line. The grand mean (mean of the means) of RPE was not different in NW compared to W (10.7 ± 1.5 in NW and 10.3 ± 1.3 in W). NW: dashed line and empty circles; W: continuous line and solid circles.

The scores of all participants for each trail segment covered either with poles (NW) or without poles (W) are plotted in Figure 5. The regression line was slightly but not significantly above the 1:1 correspondence line. The grand mean (mean of the means) of RPE was not different in NW compared to W (10.7 ± 1.5 in NW and 10.3 ± 1.3 in W). 

## 4. Discussion

This study compared the physiological and perceptual differences between NW and W when conducted in ecologically valid natural environments. This is apparently the first study to examine NW outside of a laboratory environment. In this study, the physiological response to NW was compared with that of ordinary W over long distances (several kilometres), and varying slopes and terrains in a mountain environment; the typical conditions in which this physical activity is practiced The major findings of this study were that energy expenditure and HR was higher during NW compared to W for all the conditions tested, though RPE scores did not differ under any conditions.

Rodgers et al. [6] found in laboratory experiments significant differences in energy consumption and VO_2_ (+12%) during submaximal exercise, in NW compared with ordinary W, but no difference in RPE. Porcari et al. [15] reported even greater differences in VO_2_ (+23%), HR (+16%), and energy consumption (+22%), in NW compared with W, with an RPE just slightly higher in NW than in W, but with no significant differences. Other studies have examined NW under field conditions using a track. Church et al. [16] confirmed the differences observed in laboratory studies between NW and W (i.e., higher VO_2_ and energy consumption (+20%) with invariant RPE), whilst Schiffer et al. [3] comparing NW, W and jogging confirmed the differences between NW and W, though the difference was lower than reported by others (+8%). 

The impact of the conditions of the surfaces and slope on which the subjects walk, and of the length of the poles, upon energy consumption in NW has been considered in several recent studies. Hansen et al. [7] have demonstrated that changing the length of the poles can change the energy consumption uphill, or downhill. Perrey and Fabre [25] found differences downhill, but no differences uphill. Schiffer et al. [26] have extended the comparison analysing a distance of 1200 m with different surfaces (a concrete surface, an artificial athletic track, and a natural grown soccer lawn) and have found that between concrete surface and soccer lawn there were significant differences in energy expenditure during the practice of NW. 

Many studies in the last 15 years have analysed the physiological response to NW either on a treadmill [5,6,15,16] or outdoors across short distances, 400 m [3] and 1200 m [26], and on artificial grounds [27]. Those studies have given a careful description of metabolic, cardiovascular and respiratory responses, but have been restricted to conditions which do not resemble those under which NW is typically practiced (natural mountainous or hilly environments).

The results obtained in the present study confirmed the significant increase of energy expenditure of locomotion using poles (NW) compared to locomotion without poles (W) is maintained even under ecologically valid conditions. Such an increase is on average +20%, without significant differences between uphill and downhill among subjects. An important finding of this present study is that, even across distances much longer than previously examined with variable slopes and terrain, there is a significant increase in energy expenditure with NW. As expected, the increase in energy expenditure is accompanied by increased cardiovascular demand with an average increase in heart rate of about +19%.

The skill of the participants examined in this study (recruited from certified NW instructors) was evident in the increase of stride length and in the speed of locomotion when using poles. Importantly, compared with prior studies, participants were free to self-select their speed to further enhance ecological validity. The average length of the steps increased by +15% and the average speed by 7% (from 5.5 to 5.9 km/h or 1.53 to 1.64 m/s). It is generally assumed that freely chosen W speed can be shown to correspond to an optimum energy efficiency [28]. The latter data require further explanation. It is widely accepted that energy cost of walking increases in proportion with speed [29]. Thus, the increase in speed by 7% implies that energy cost of locomotion is increased in proportion. This suggests that the overall increase by 20% of energy expenditure of locomotion may be explained by two components: ~7% due to the increased speed and the other of ~13% due to the specific movement of NW. This second component is likely due mainly to the use of the poles and subsequent involvement of upper limbs, in particular greater muscle activation during forward swing of the poles [30]. In accordance with previous studies [5,6,31], the increase of energy expenditure is well tolerated as indicated by the similar RPE in both NW and W.

It is well known that an increase in walking speed causes an increase in metabolic power. A difference in stride frequency higher than 10% compared with the freely chosen stride frequency can cause a higher metabolic power [32,33]. In our experiments, the speed was higher in NW, but stride frequency was within the 10% range and would not affect metabolic power. In a recent paper of Pellegrini et al. [34], the speed was kept constant and subjects were asked to walk with or without poles on a treadmill with 0 gradient. NW metabolic power was higher than W and stride frequency was lower in NW. A higher additional internal work (due to poles) was found, but the authors did not explain all the difference in metabolic cost with this extra work, instead they used the greater muscular activity (also co-contraction). These findings are similar to ours and it is likely that the additional muscular activity of the upper body explains the additional metabolic cost with NW. 

As stated in the material and methods, we had a calibration session where we found a good agreement between the armband and expired gas analyser while subjects walked (with and without poles) at a range of speeds and gradients similar to the experimental session. It should be noted, however, that this is an ecological study and subjects freely choose their preferred walking speed potentially affecting the findings.

## 5. Conclusions

NW has become in recent years a widespread open-air physical activity but there is lack of data on the physiological stimuli induced under ecologically valid conditions. The present study provides a first account of the physiological and perceptual response to NW compared to W in the environmental conditions where such activity is generally practised. These results obtained by our group were consistent with previous findings in more controlled environments (treadmill, artificial indoor or outdoor tracks). Taken together our data suggests that NW could be an important tool for an increase of caloric expenditure or for mild aerobic training, without any significant differences in perceived exertion.
